# Assessment of Sleep Quality Among Adolescents and Adults With Self-Diagnosed Irritable Bowel Syndrome, in Jeddah, Saudi Arabia

**DOI:** 10.7759/cureus.42778

**Published:** 2023-07-31

**Authors:** Aisha A Alghamdi, Ahmed M Alghamdi, Murooj A Alshareef, Abdulrahman A AlGhamdi, Rahaf A Alghamdi, Alyah A AlAmri, Ghaday T Alzahrani

**Affiliations:** 1 Medicine, King Abdulaziz University, Jedaah, SAU; 2 Medicine, King Abdulaziz University, Jeddah, SAU

**Keywords:** s: irritable bowel syndrome, jeddah, ibs, adults, adolescents, quality, sleep

## Abstract

Background

The frequency of irritable bowel syndrome (IBS) has increased significantly in the last ten years. Few studies were done in Saudi Arabia to assess the relationship between sleep quality and IBS. This study aims to assess the prevalence of IBS and its association with sleep quality among adolescents and adults in Saudi Arabia.

Methods

A cross-sectional study was done on 651 participants aged 15-65 years. An online questionnaire was used to collect data, including demographics; the Rome IV criteria (R4DQ) was used in the diagnosis of IBS, the IBS symptoms severity scale (IBS-SSS) assessed IBS symptoms and severity, and the Pittsburgh Sleep Quality Index (PSQI) was used to assess sleep quality.

Results

Based on the Rome IV criteria, 25.7% of the participants had IBS. Among them, 23.3%, 17.9%, 47.3%, and 11.5% had IBS types constipation (C), diarrhea (D), mixed bowel habits (M), and undefined subtype (U), respectively. Mild, moderate, and severe IBS were found among 43.1%, 39.5%, and 17.4% of IBS cases, respectively. About 46% had poor sleep quality, which was significantly higher among those with younger mean age, females, and students. Patients with IBS exhibited a considerably greater prevalence of poor sleep quality, and IBS-C had the highest prevalence.

Conclusion

A correlation was found between poor sleep quality and the positive status of IBS diagnosis among adolescents and adults. An evaluation of specific sleep disorders among IBS patients is needed.

## Introduction

Irritable bowel syndrome (IBS) is a chronic gastrointestinal condition characterized by periodic, chronic abdominal discomfort without underlying organic lesions as verified by endoscopic, laboratory, or radiographic investigations. IBS has a complex etiology that is not well understood; its development may have been influenced by several factors, including genetic susceptibility, altered gut-brain connections, visceral hypersensitivity, mucosal inflammation, and bowel microbial variation [1, 2]. It can affect people of all ages and from various economic, psychosocial, and racial backgrounds [2, 3]. IBS was found to be most common in people between the ages of 20 and 40 [4]. Furthermore, numerous publications state that women are more likely than men to have IBS, that hereditary factors can influence the syndrome, and that up to 30% of people may develop the condition as a result of their family history [5].

IBS affects 10 to 25% of the world's population [6]. Furthermore, its signs and symptoms are among the most common reasons for primary care visits [7]. However, 2.4-3.5 million people in the United States alone seek medical attention for IBS each year. IBS prevalence was recently reported to be 9.2 percent in 53 surveys conducted in 38 countries involving 395,385 people [8].

According to a review of the literature, the prevalence has risen, with rates ranging from 8.9 to 31.8% in the world [9], with the frequency increasing significantly in the last ten years, particularly in Saudi Arabia. IBS is an annoyance that should not be underestimated [10].

Due to the lack of objective diagnostic findings, IBS can currently only be diagnosed based on a patient's personal history. The Rome IV criteria are currently used to diagnose IBS because they include the following elements: recurrent abdominal pain, at least one day per week for the last three months, with a symptom onset of at least six months before the diagnosis [5, 11, 12].

Patients are typically classified as having IBS-C (predominant constipation), IBS-D (predominant diarrhea), or IBS-M if their abdominal pain is accompanied by either diarrhea or constipation, or both (IBS with mixed bowel habits). If the symptoms do not clearly fall into one of these three groups, the diagnosis is given as IBS-U (unclassified) [13].

Sleeping accounts for roughly one-third of a person's life and is one of the body's biological activities [14]. Sleep deprivation has an impact on the body's physiological processes, including the neurological, metabolic, and endocrine systems [15]. Sleep deficiency, which affects an estimated 37.6% of the IBS population, is a well-known health issue. The comprehensive concept of sleep deficiency includes poor sleep quality and sleep deprivation [16]. Furthermore, between 7.1% and 73.9% of IBS patients have sleep problems, and there may be a link between sleep problems and more severe gastrointestinal symptoms [17].

A few regional studies were done on this subject, which were limited by the decreased number of participants. Thus, this study aimed to assess the prevalence of irritable bowel syndrome and its association with the quality of sleep among adolescents and adults in Saudi Arabia.

## Materials and methods

Study design, setting, and time

A cross-sectional study was done in Saudi Arabia over three months.

Study participants

Six hundred fifty-one Saudi residents were the participants of the present study. The inclusion criteria included participants aged 15-65 years, who agreed to participate in the study. And the exclusion criteria focused on excluding participants who refused to participate, were diagnosed with inflammatory bowel disease, diagnosed with any clinical psychiatric illness, and had incomplete responses.

Data collection

A Google Forms (Google, Mountain View, California) link to the study's questionnaire, which was written in two languages, Arabic and English, was sent to participants via social media platforms, and contained consent to use data for research. All participants were given informed consent as required by University Ethics Committee for cross-sectional studies. The questionnaire consisted of four sections. The first was to collect demographic data. The second included the Rome IV criteria (R4DQ), which was used in the diagnosis of IBS among the studied population; two versions of two languages, Arabic and English, were obtained from the Rome Foundation [18]. Thethird section included the IBS symptoms severity scale (IBS-SSS) to assess IBS symptoms and severity, also in the two languages Arabic and English, which was also obtained from the Rome Foundation [19]. The fourth included the Pittsburgh Sleep Quality Index (PSQI) to assess sleep quality, the English version was obtained from Buysse et al. [20], and AlMaqbali et al.'s validation of the Arabic translation was utilized as well [21]. The PSQI results were classified into good and poor according to Fabbri et al., which categorized participants who scored five and above in the global PSQI score as poor sleepers [22].

Data analysis

Data was analyzed using SPSS version 26 (IBM Inc., Armonk, New York). The Chi-squared test (χ2) and the Kendell Tau were applied to qualitative data that was expressed as numbers and percentages to examine the relationship between the variables. The Mann-Whitney and Kruskal-Wallis tests were used to analyze non-parametric variables, and quantitative data was presented as median and inter-quartile range (IQR). A p-value of less than 0.05 was regarded as statistically significant.

## Results

Table 1 shows that the median age of studied participants was 28 years, 72.7% were females, 73.1% had a bachelor's degree in education, and 39.3% were students. About 45% (45.2%) had a monthly income <5000 SR, and 18.4% had chronic diseases.

**Table 1:** Distribution of studied participants according to their demographic characters and chronic disease presence (N=651)

Variable	N (%)
Age in years (median)	28
Gender	
Female	473 (72.7)
Male	178 (27.3)
Nationality	
Non-Saudi	25 (3.8)
Saudi	626 (96.2)
Education	
Primary school	2 (0.3)
Middle school	3 (0.5)
Secondary school	115 (17.7)
Bachelors	476 (73.1)
Above Bachelors	53 (8.1)
Master	2 (0.3)
Employment	
Government employee	197 (30.3)
None	83 (12.7)
Private sector employee	53 (8.1)
Retired	50 (7.7)
Self-employed	12 (1.8)
Student	256 (39.3)
Monthly income	
<5000 SR	294 (45.2)
5000-10000 SR	122 (18.7)
10000-20000 SR	191 (29.3)
>20000 SR	44 (6.8)
Chronic diseases	
No	531 (81.6)
Yes	120 (18.4)

**Figure 1:**
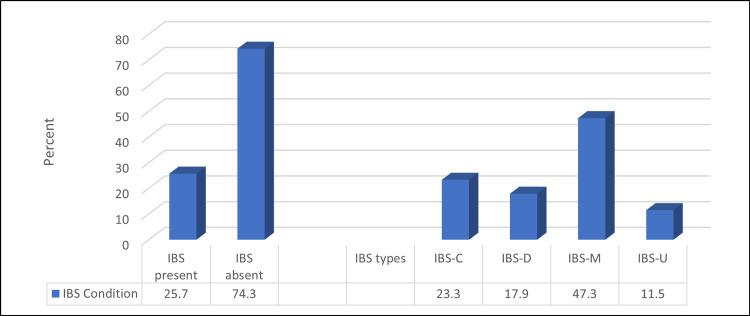
Percentage distribution of studied participants according to IBS prevalence and its types (N=651) IBS - irritable bowel syndrome

Based on the scoring algorithm for the Rome IV Diagnostic Questionnaire for Adults, 167 (25.7%) of the participants had IBS. Among them, (No.:167), 39 (23.3%), 30 (17.9%), 80 (47.3%), and 18 (11.5%) had IBS types C, D, M, and U, respectively (Figure 1).

**Figure 2:**
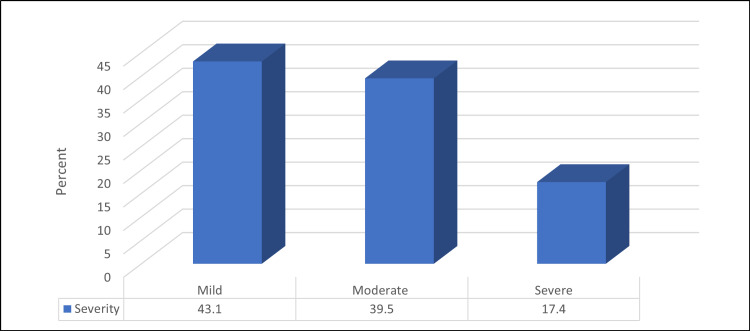
Percentage distribution of studied IBS participants according to its severity (N=167) IBS - irritable bowel syndrome

Among the IBS patients, mild severity had the highest percentage, while severe was the lowest; 72 (43.1%) and 29 (17.4%), respectively (Figure 2).

**Figure 3:**
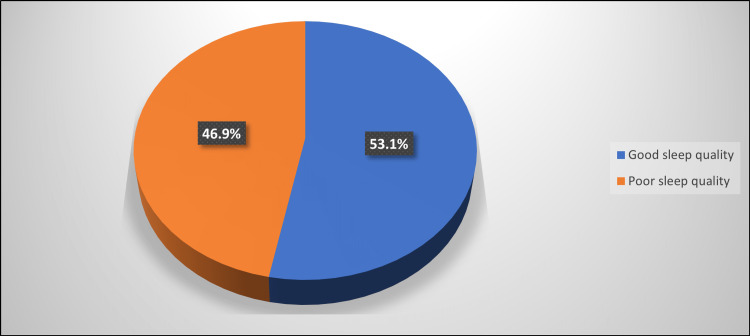
Percentage distribution of studied participants according to sleep quality (N=651)

The median PSQI for all participants was 5, and 46.9% had poor sleep quality (Figure 3).

Table 2 demonstrates that participants who had IBS were significantly of Saudi nationality (p=<0.05). A non-significant relationship was found between IBS prevalence and other demographics or chronic disease status (p=>0.05).

**Table 2:** Relationship between IBS prevalence and participants' demographics and chronic disease presence (N=651) IBS - irritable bowel syndrome

Variable	IBS		χ2	p-value
	No N (%)	Yes N (%)		
Age in years, median (IQR)	29 (25)	25 (18)	1.5	0.133
Gender				
Female	350 (72.3)	123 (73.7)	0.11	0.738
Male	134 (27.7)	44 (26.3)		
Nationality				
Non-Saudi	23 (4.8)	2 (1.2)	4.24	0.039
Saudi	461 (95.2)	165 (98.8)		
Education				
Primary school	2 (0.4)	0 (0.0)	2.33	0.802
Middle school	2 (0.4)	1 (0.6)		
Secondary school	89 (18.4)	26 (15.6)		
Bachelors	349 (72.1)	127 (76)		
Above Bachelors	40 (8.3)	13 (7.8)		
Master	2 (0.4)	0 (0.0)		
Employment				
Government employee	148 (30.6)	49 (29.3)	2.82	0.727
None	57 (11.8)	26 (15.6)		
Private sector employee	39 (8.1)	14 (8.4)		
Retired	40 (8.3)	10 (6)		
Self-employed	10 (2.1)	2 (1.2)		
Student	190 (39.3)	66 (39.5)		
Monthly income				
<5000 SR	216 (44.6)	78 (46.7)	0.43	0.934
5000-10000 SR	91 (18.8)	31 (18.6)		
10000-20000 SR	145 (30)	46 (27.5)		
>20000 SR	32 (6.6)	12 (7.2)		
Chronic diseases				
No	392 (81)	139 (83.2)	0.41	0.519
Yes	92 (19)	28 (16.8)		

Table 3 shows that poor sleep quality was significantly higher among participants with younger median age, females, and students (p=<0.05).

**Table 3:** Relationship between sleep quality and participants' demographics and chronic diseases presence (N=651)

Variable	Sleep quality		χ2	p-value
	Good N (%)	Poor N (%)		
Age in years, median (IQR)	34.6 (24)	25 (21)	3.36	0.001
Gender				
Female	238 (68.8)	235 (77)	5.57	0.018
Male	108 (31.2)	70 (23)		
Nationality				
Non-Saudi	13 (3.8)	12 (3.9)	0.01	0.907
Saudi	333 (96.2)	293 (96.1)		
Education				
Primary school	1 (0.3)	1 (0.3)	6.51	0.259
Middle school	0 (0.0)	3 (1)		
Secondary school	54 (15.6)	61 (20)		
Bachelors	258 (74.6)	218 (71.5)		
Above Bachelors	32 (9.2)	21 (6.9)		
Master	1 (0.3)	1 (0.3)		
Employment				
Government employee	118 (34.1)	79 (25.9)	15.95	0.007
None	46 (13.3)	37 (12.1)		
Private sector employee	31 (9)	22 (7.2)		
Retired	32 (9.2)	18 (5.9)		
Self-employed	7 (2)	5 (1.6)		
Student	112 (32.4)	144 (47.2)		
Monthly income				
<5000 SR	141 (40.8)	153 (50.2)	7.08	0.069
5000-10000 SR	66 (19.1)	56 (18.4)		
10000-20000 SR	115 (33.2)	76 (24.9)		
>20000 SR	24 (6.9)	20 (6.6)		
Chronic diseases				
No	286 (82.7)	245 (80.3)	0.58	0.444
Yes	60 (17.3)	60 (19.7)		

**Figure 4:**
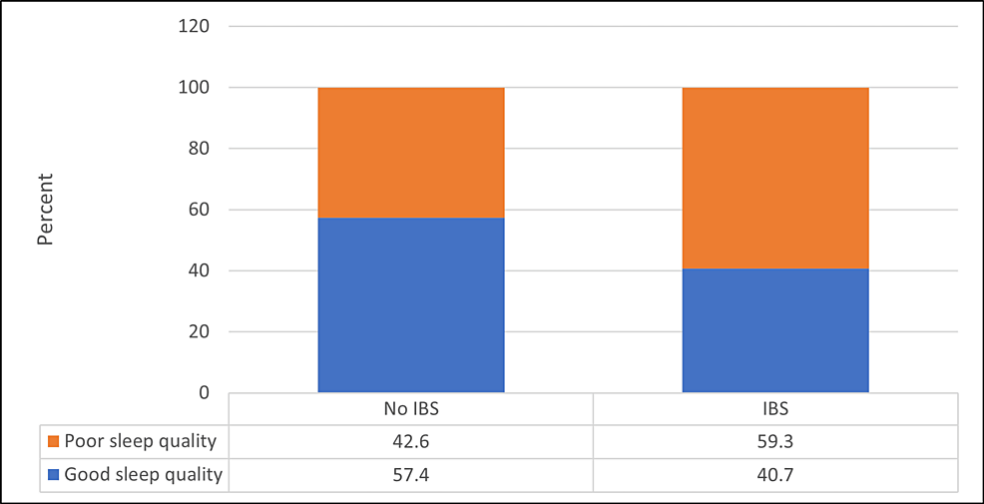
Relationship between IBS prevalence and sleep quality (N=167) χ2 = 13.93, p-value = <0.001 IBS - irritable bowel syndrome

Figure 4 shows that IBS patients were a significantly higher percentage of those having poor sleep quality compared to non-IBS patients (p=<0.05). It was found that the mean PSQI score for participants with IBS was 6.92 ± 3.35 compared to those having no IBS (5.37 ± 2.95).

**Figure 5:**
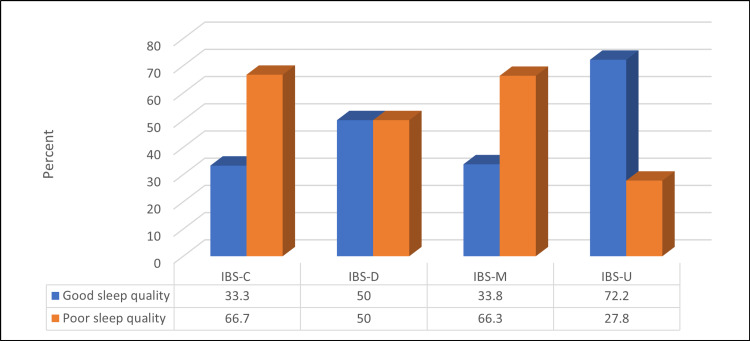
Relationship between IBS types and sleep quality (N=167) Kendell Tau = 0.09, p-value = 0.18 IBS - irritable bowel syndrome

Figure 5 shows that a non-significant relationship was found between IBS status and sleep quality (p=>0.05).

**Figure 6:**
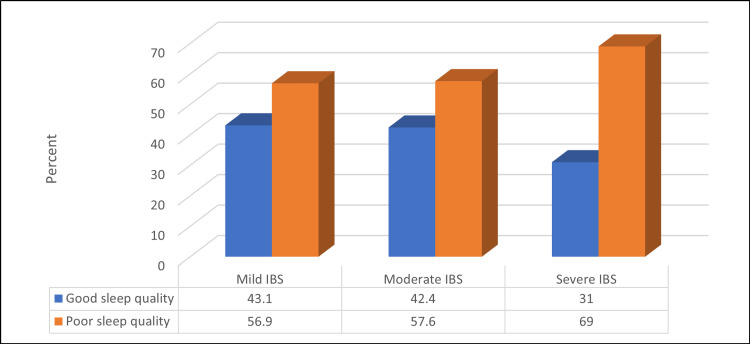
Relationship between IBS severity and sleep quality (N=167) Kendell Tau = 0.1, p-value = 0.06 IBS - irritable bowel syndrome

Figure 6 illustrates a non-significant relationship between IBS severity and sleep quality (>0.05).

The mean PSQI score for mild, moderate, and severe IBS was 6.23 ± 3.09, 6.36 ±3.41 and 7.68± 3.49, respectively, with a non-significant difference (Kruskal Wallis test = 4.63, p-value = 0.098). 

## Discussion

The goal of this study was to see how common irritable bowel syndrome was and how it related to sleep quality among Saudi adolescents and adults.

The Rome IV Diagnostic Questionnaire Scoring Algorithm revealed that 25.7% of the participants had IBS. The prevalence of IBS found in the current study is similar to that found in a recent study of female secondary school students in Ar Rass, Qassim region, Saudi Arabia. According to the study, the prevalence was 21.4% [23]. This prevalence, however, is higher than that reported in a Korean study of adolescents, which found it to be 10.7% [24]. It is also higher than the Rome III criteria reported among Japanese adolescents (18.6%) [25]. And higher than the 2.3 percent found among Colombian adolescents using the same Rome IV criteria [26]. This variation in IBS prevalence could be attributed to differences in diagnosis rates based on whether the Rome III or Rome IV criteria were used; the former used the criteria of pain or discomfort, whereas the latter depended on pain alone with the removal of discomfort criterion.

The current study's prevalence corresponds to the prevalence of IBS in other countries, with 38.4% of Indonesian adolescents meeting the Rome III criteria. Other studies [27] have found a high prevalence of IBS among adolescents. Using ROME III criteria, Soares et al. discovered a prevalence of 32.2% for IBS [28]. Globally, the prevalence of IBS among people aged 15 and up is 8.8-14% [29], but it is higher among adolescents, at 16.5-38.4% [25, 27].

Type C (23.3%), D (17.9%), M (47.3%), and U (11.5%) IBS were identified in this study. This finding differs from a previous Saudi study, which found that the most common IBS type was IBS-M [30]. Alharbi et al. used Rome IV criteria to investigate the most common subtypes in the northern Saudi population. They also discovered that IBS-M was the most common subtype, followed by IBS-C, IBS-D, and IBS-U [31]. In a separate study of nurses at King Abdulaziz University Hospital in Jeddah, Saudi Arabia, the IBS-M was the most common (54.5%), followed by IBS-C (27.3%), IBS-U (12.1%), and IBS-D (6.1%) [29].

According to the current study, 43.1%, 39.5%, and 17.4% of IBS patients had mild, moderate, or severe IBS, respectively. In the Ibrahim et al. study, 18.2% were in remission, while the remaining 66.7, 12.1, and 3.0% had mild, moderate, or severe IBS, respectively [32].

In terms of sleep quality, the current study discovered that 46.9% of participants had poor sleep quality, with participants with a younger mean age, females, and students having a significantly higher prevalence of poor sleep quality. Previous Saudi studies on university students revealed a high prevalence of poor sleep quality, with the majority, 63.9%) [33] and 64.4% [34], having poor sleep quality.

Participants with younger mean age, females, and students were significantly more likely to have poor sleep quality. The higher prevalence of poor sleep quality among female participants was also revealed in a previous study, where female students were found to be more likely than male students to experience sleep disturbances [35]. Another study discovered that women were more susceptible to insomnia [36]. Other studies, however, found no gender difference [27].

Many studies have investigated the relationship between gastrointestinal physiology and inflammation, and sleep and found a significant correlation. A strong link was found between the pathophysiological mechanisms of insomnia and IBS [25, 27].

The current study found that IBS patients had a significantly higher percentage of poor sleep quality. An Indonesian study found the same link, with 53.1% of those diagnosed with IBS having sleep disorders [27]. In other studies, the prevalence of IBS among adolescents was found to increase the incidence of insomnia by a factor of 3.3 [24]. In addition, sleep problems were significantly associated with IBS in a study of Japanese adolescents, and IBS was associated with a delay in sleep onset in adolescents, which is a symptom of insomnia [25]. A systematic review of the relationship between IBS and sleep problems found a strong link between the two [16].

Previous research has identified IBS to be associated with sleep disorders. Adolescents with IBS frequently experience difficulty sleeping, frequent awakening, and difficulty returning to sleep after awakening. The high prevalence of IBS in adolescents, as well as the high prevalence of sleep disorders in IBS, have a negative impact on quality of life and disrupt physical development, behavior, and learning achievement [27]. Noor et al., Young et al., and AbdAllah et al. also discovered the same link between IBS and poor sleep quality [27, 37, 38].

It has been proposed that the relationship between IBS and poor sleep quality is a vicious cycle linked to the gut-brain axis [39]. Changes in sleep patterns can cause leukocytosis and an increase in natural killer cells, as well as an increase in inflammatory cytokine production, such as TNF and IL6. It's worth noting that cytokines that regulate sleep and wakefulness are also involved in the pathogenesis of IBS [40].

Among those reported mild and moderate severity of symptoms on the IBS severity scoring system (IBS-SSS), the sleep quality discrepancy was of a non-significant difference. As shown by percentages, a comparison of good and poor sleep qualities amongst the subdivision of the IBS-SSS in Figure 6. Whereas, in severe cases, the difference is 31% to 69% (p-value = 0.06), which illustrates that the patients having a severe form of IBS and poor sleep quality is more than twice those having a severe form of IBS and good sleep quality. Basharat et al. discovered a link between the severity of IBS symptoms and sleep disruptions [41].

According to an Egyptian study, there is a significant relationship between sleep quality and IBS severity, as well as a significant positive correlation between the PSQI global score and the IBS-SSS. The PSQI global score had a significant positive correlation with both FSS and IBS-SSS. According to the same study, mild and moderate IBS increased the risk of poor sleep quality by 12.97 and 4.71 folds, respectively [38].

Limitations

The use of a cross-sectional study design that could reveal the association between variables without determining the causal relationship was a limitation of the current study. Other potential confounders for the association could include the presence of a mood disorder; the lack of this data point does limit the study. Another limitation is that IBS and sleep quality are both self-reported, and no objective tests could be used for assessment. Lastly, the sampling using a confidence interval of 95% could be increased to 99% with a margin error of 1% instead of 5%. Therefore we advise our future fellow researchers to try to increase the sample size, as this may contain more accuracy in terms of results to ensure the diversity of participants.

## Conclusions

In this study, the prevalence of IBS was 25.7% of the participants using the Rome IV criteria, and 46.9% had poor sleep quality. Participants with younger mean age, females, and students had a significantly higher prevalence of poor sleep quality. And IBS patients had a significantly higher percentage of poor sleep quality, with IBS-C having the highest prevalence.

In the management of IBS and poor sleep quality, a relational approach to IBS and poor sleep is suggested. IBS patients should be screened for poor sleep quality using a quick method such as the PSQI. And those with abnormal results should undergo additional testing.

We also believe that there are medical conditions that have a direct impact on the quality and efficiency of sleep, and from our perspective, in order for the irritable bowel patient to experience high-quality sleep, we stress the need to exclude medical diseases associated with a direct effect on sleep quality, such as obstructive sleep apnea, or insomnia, whether it is primary or secondary, and this is done under the supervision of the specialist doctor who diagnosed the patient with the functional bowel condition, i.e., to expand the research to find a direct causal relationship between IBS and a specific sleep disorder of the sleep disorders spectrum.
